# Popliteal Cyst Compressive Tibial Neuropathy and Venous Insufficiency: A Case Report

**DOI:** 10.7759/cureus.53499

**Published:** 2024-02-03

**Authors:** YuChia Wang, Haley E Berry, Ryan J Froom, Kendall Couch, Daniel M Kopolovich, Jonathan A Godin

**Affiliations:** 1 Orthopaedic Surgery, Steadman Philippon Research Institute, Vail, USA; 2 Orthopaedic Surgery, The Steadman Clinic, Vail, USA

**Keywords:** compressive neuropathy of the peripheral nerves, peripheral edema, tibial nerve palsy, popliteal cyst, knee injuries

## Abstract

Popliteal cysts are a collection of synovial fluid found in the popliteal fossa that typically form in adults in association with traumatic injuries, degenerative conditions, or inflammatory arthritis of the knee. While often asymptomatic, popliteal cysts may become problematic as enlarging and ruptured cysts may compress surrounding neurovascular structures, resulting in lower extremity edema or peripheral neuropathy. We report a unique case of a symptomatic popliteal cyst in a patient with both compressive neuropathy and venous congestion in the setting of a non-ruptured popliteal cyst after a surgically repaired intraarticular injury. Magnetic resonance imaging (MRI) showed a synovial cyst abutting the posterior neurovascular bundle and evidence of avascular necrosis. An open posterior cyst decompression was done, and the patient was able to report significant symptomatic improvement over the course of two weeks postoperatively. The previously noted varicose veins also demonstrated noticeable resolution. While relatively common, popliteal cysts may require prompt surgical decompression in order to provide effective symptomatic relief.

## Introduction

A popliteal cyst is a collection of synovial fluid traditionally located in the gastrocnemius-semimembranosus bursa in the posterior aspect of the knee [1,2]. These usually form due to a weakening of the synovial capsule in this area and a lack of anatomic support [3]. A popliteal cyst can present as a palpable mass in conjunction with prior trauma or degenerative knee pathologies. Any intra-articular process with an effusion can lead to distension of the capsule and posterior bursa through a one-way valve. Most commonly, these intra-articular processes include meniscus tears, chondral damage, or ligamentous injuries. While popliteal cysts are often asymptomatic and incidental findings, they can cause symptoms such as swelling, decreased range of motion, and pain that requires treatment. Sansone et al. found that 4.7% of patients between the ages of six to 89 years with knee pain had a popliteal cyst [4]. In rare instances, the mass effect from a popliteal cyst can lead to compression of the surrounding neurovascular structures, resulting in neuropathy and/or venous congestion. 

Surgical excision of popliteal cysts may be indicated when symptoms persist despite non-operative management and after treatment of any underlying intraarticular pathology. Open and arthroscopic surgical treatments have both been described as viable options for decompression of popliteal cysts and both have good success rates in preventing recurrence. Arthroscopic cystectomy with valve excision recurrence rate is reported at 12.4% [5]. Saylik et al., on the other hand, reported a popliteal cyst recurrence rate of 1.94% in their series after open cyst excision with stalk closure [6].

A posterior open approach can be utilized as a primary method for cyst excision or for cases in which the symptoms are still present after the intra-articular pathology has already been addressed and/or when arthroscopic excision fails. Care must be taken to properly identify underlying pathologies and protect neurovascular structures to avoid potential iatrogenic complications [1,2,7].

Our case is the first to the authors' knowledge to describe a posterior tibial neuropathy with concomitant venous congestion secondary to popliteal cyst compression of the neurovascular bundle and subsequent avascular necrosis of the femoral condyle and fibular head. 

## Case presentation

The patient is an healthy 55-year-old male presenting with persistent unilateral knee pain, tibial neuropathy, and mild/moderate venous insufficiency status post arthroscopic lateral meniscal repair at an outside institution. The knee discomfort originated from a skiing injury sustained two years prior to presentation to our institution. At the time, the patient was diagnosed with a proximal lateral gastrocnemius tendon tear and was managed non-operatively with physical therapy. Over the following year, the patient developed worsening swelling throughout the right leg distal to the knee joint as well as a feeling of fullness in the posterior aspect of his knee without symptomatic relief with other non-operative treatment modalities. Magnetic resonance imaging (MRI) showed a lateral meniscus tear with concerns for possible avascular necrosis of the proximal femur and fibular head. The patient then underwent a right knee arthroscopic all-inside lateral meniscal repair as well as bone marrow aspirate concentrate (BMAC) injection into the lateral femoral condyle.

One year after the patient’s initial surgery, he presented to our clinic reporting minimal pain relief from the previous surgery. On physical examination, the patient continued to have posterior fullness to the knee that was tender to palpation. He also presented with a decreased range of motion measuring 7-112 degrees, significantly decreased from his contralateral side, which measured 0-135 degrees. Numerous varicose veins and unilateral lower extremity edema were also noticeable, though dorsalis pedis and posterior tibial pulses were palpable. Neurologically, the patient reported decreased sensation along the bottom of the foot in the tibial nerve distribution but had relatively normal motor function. 

**Figure 1:**
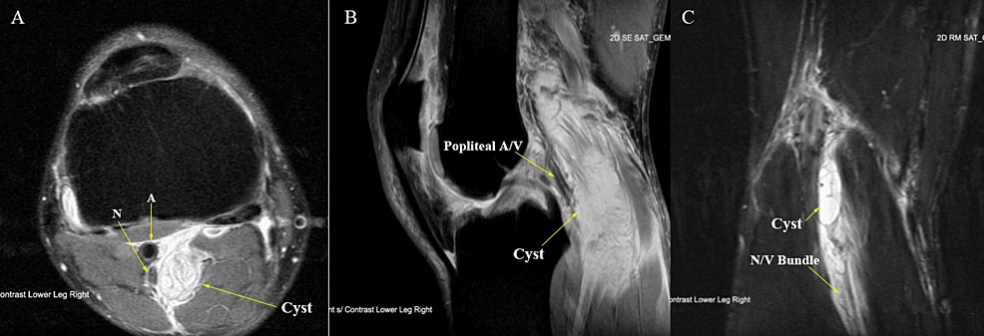
Magnetic resonance imaging in the axial (A), sagittal (B), and coronal (C) planes showing the close proximity and compression of the popliteal cyst against the posterior neurovascular structures. A: Artery; N: Nerve; A/V: Artery and Vein; N/V: Neurovascular; Cyst: Popliteal Cyst of Interest.

MRI of the right knee demonstrated a 25 x 15 x 18 mm synovial cyst posterior to the tibial insertion of the posterior cruciate ligament abutting the posterior neurovascular bundle with increased dilation of the vein distal to the cyst (Figure 1). The MRI also showed a tear of the lateral head of the gastrocnemius muscle in the proximal leg with edema in the surrounding area. In addition, there was severe bone marrow edema consistent with avascular necrosis of the lateral femoral condyle, fibular head, and lateral aspect of the patella. Venous and arterial Doppler ultrasounds were within normal limits. 

**Figure 2:**
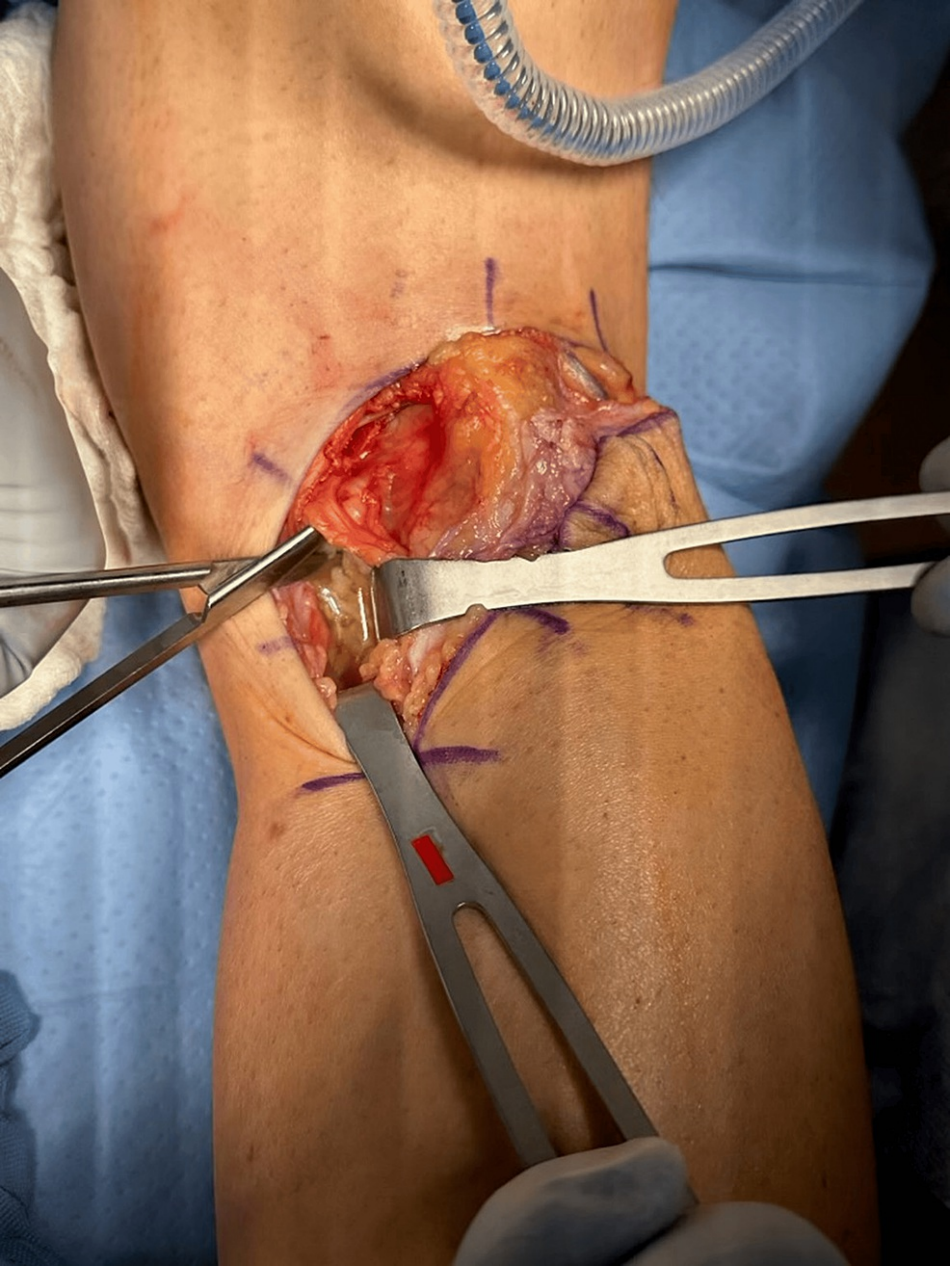
Open posterior approach to the knee. Army-Navy retractors are used to retract the medial gastrocnemius tendon medially and blunt dissection is carried deep to this to expose and decompress the cyst.

**Figure 3:**
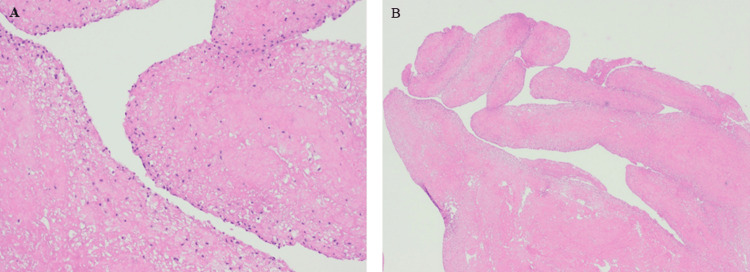
Histopathologic slides at 4x (A) and 2x (B) magnification showing fibrinoid necrosis and synovial fronds (Hematoxylin and eosin stain).

The patient’s symptoms were consistent with compression along the posterior neurovascular bundle secondary to the popliteal mass. Due to persistent symptoms despite prior conservative and operative treatments, the patient was indicated for an open posterior cyst decompression (Figure 2). An inverted L-shaped incision was made along the posterior knee with the horizontal limb in the knee crease and the longitudinal limb extending distally along the medial side of the leg. Hemostasis was achieved and dissection was carried through the subcutaneous tissue to the underlying fascia. This was then incised sharply in line with the skin incision. The interval between the semimembranosus and the medial head of the gastrocnemius tendon was then developed via blunt dissection to avoid injury to the surrounding neurovascular structures. This exposed the cystic structure, which was subsequently decompressed. The collected specimen was sent for pathology and showed extensive fibrinoid necrosis and synovial fronds, as shown in Figure 3.

At the two-week follow-up, the patient reported significant improvements in his knee pain and lower extremity paresthesia. On examination, there was noticeable resolution to the previously noted varicose veins and posterior knee swelling. 

## Discussion

Popliteal cysts most commonly develop along the posteromedial aspect of the knee between the medial head of the gastrocnemius and the semimembranosus tendon due to a lack of structural support in this area. The cystic fluid is normally confined to the space of the semimembranosus bursa, preventing its extension laterally and protecting the posterior neurovascular bundle. On rare occasions, however, the cyst does extend laterally and causes neural or vascular symptoms, or combination thereof as a result of structural compression secondary to the enlarging cyst. The tibial nerve is more commonly compromised in this situation given its anatomic relationship to the semimembranosus bursa. The nerve is the most medial and superficial structure in the neurovascular bundle [8]. Patients with tibial neuropathy can present with paresthesias along the sole of the foot, posterior knee pain, or gastrocnemius atrophy. 

Lower extremity venous insufficiency as a result of symptomatic popliteal cysts may also occur. As the cyst continues to expand, the popliteal artery or vein can be compressed. The clinical presentation may resemble that of deep vein thrombosis (DVT) with lower extremity edema, erythema, and pain. Ruptured popliteal cysts can further complicate the clinical presentation due to overlapping symptoms of pain, swelling, and redness in the calf muscle. A positive Homan’s sign can be elicited even in the absence of a DVT with a ruptured popliteal cyst. In very rare instances, ruptured popliteal cysts can lead to compartment syndrome and subsequent neurovascular compromise [9].

Proper diagnostic testing is crucial for delineating the underlying cause, as treatment options can vary greatly between different diagnoses. The rarity of tibial nerve palsy, especially with vascular compromise as a complication of popliteal cysts, underscores the need for careful diagnostic and therapeutic strategies. Clinicians should maintain a high index of suspicion for such cysts in patients with unexplained neuropathic or vascular symptoms in the lower extremities.

This case contributes to the spectrum of presentations and outcomes associated with popliteal cysts. While arthroscopic techniques have been successful, open surgical techniques remain a viable option, especially when arthroscopic measures have not provided relief [10-13]. This case reinforces the importance of individualized treatment planning and highlights the potential for significant improvement with the appropriate surgical technique. This case adds valuable data to the growing body of evidence supporting tailored surgical intervention in the management of symptomatic popliteal cysts with neuropathic and vascular complications. 

## Conclusions

The management of popliteal cysts, particularly when presenting with compressive neuropathy or venous congestion, is a clinical challenge as evidenced by the present report. Surgical intervention via open posterior cyst decompression yielded significant improvement in the patient's symptoms, a notable outcome reflecting the efficacy of direct surgical approaches in such cases​​. 
